# Sputnik virophage disrupts the transcriptional regulation of its host giant virus

**DOI:** 10.1128/jvi.00192-25

**Published:** 2025-03-12

**Authors:** Jingjie Chen, Hiroyuki Ogata, Hiroyuki Hikida

**Affiliations:** 1 Bioinformatics Center, Institute for Chemical Research, Kyoto University 26337 , Uji, Japan; Michigan State University, East Lansing, Michigan, USA

**Keywords:** virophage, giant virus, transcriptome analysis, eukaryotic virus, viruses of microorganisms, virus-virus interaction, gene regulation

## Abstract

Sputnik virophages are small double-stranded DNA (dsDNA) viruses that replicate only inside host amoebae infected with giant dsDNA viruses, mimiviruses. Sputnik infection affects mimivirus replication, but their molecular interaction remains poorly understood. Here, we performed a time-course transcriptome analysis of *Acanthamoeba castellanii* cells infected with Acanthamoeba polyphaga mimivirus (APMV; hereafter referred to as Sputnik^−^ cells) and those infected with both APMV and Sputnik 3 virophage (Sputnik^+^ cells). The gene expression patterns of the amoeba were similar between these two conditions, whereas the expression of APMV genes was drastically affected by Sputnik, depending on the timing of their expression. Early-expressed APMV genes showed similar expression patterns in both conditions at the early stage of infection. However, at later stages, their expression levels remained higher in Sputnik^+^ cells than in Sputnik^−^ cells, suggesting a prolongation of early gene expression by Sputnik. Late-expressed APMV genes showed lower expression at earlier stages in Sputnik^+^ cells, but their expression levels reached or exceeded those in Sputnik^−^ cells at later stages, indicating a delay in gene expression. Overall, our results demonstrated that Sputnik infection drastically alters the transcriptome of APMV rather than amoeba, likely by disturbing the transition from early to late stages of APMV infection.

**IMPORTANCE:**

Virophages are small double-stranded DNA (dsDNA) viruses parasitizing other dsDNA viruses, such as giant viruses. Virophages inhibit the replication of giant viruses, and some protists use virophages as a defense system against giant viruses. However, molecular interactions among host cellular organisms, giant viruses, and virophages are largely unknown. Here, we performed a time-course transcriptome analysis of *Acanthamoeba castellanii* cells infected with Acanthamoeba polyphaga mimivirus (APMV) and those infected with both APMV and Sputnik 3 virophage. We demonstrated that the virophage has little effect on the amoeba transcriptome and primarily hijacks the transcriptional machinery of the giant virus. Furthermore, virophage infection alters giant virus gene expression, depending on their expression timing. The expression of early genes was prolonged, while that of late genes was delayed, suggesting that virophage infection disrupts the transition from the early to late stages of giant virus infection. This study provides molecular insights into the interactions within this unique tripartite system.

## INTRODUCTION

Virophages are double-stranded DNA (dsDNA) viruses associated with other dsDNA viruses, nucleocytoviruses, which have large genomes and particles and are often called giant viruses (1–3). The first virophage, Sputnik, has an 18 kbp genome in 80 nm particles and is associated with a giant virus, mimivirus, which has a 1.2 Mbp genome in 700 nm particles. Sputnik and mimiviruses infect free-living amoebae of the genus *Acanthamoeba*, but Sputnik cannot replicate without mimivirus infection. Furthermore, Sputnik infection reduces the infectivity of progeny mimiviruses, leading to the concept of “a virus parasitizing another virus” and the designation of virophage (4).

Following the discovery of Sputnik, related viruses have been isolated with mimiviruses and other nucleocytoviruses (1, 5), such as Mavirus associated with Cafeteria roenbergensis virus (CroV) (6) and Gezel-14T associated with Phaeocystis globosa virus-14T (PgV-14T) (7). In addition to the virophages isolated with these giant viruses, sequences of virophage relatives have been identified in various environmental metagenomic data (8–10), ranging from aquatic environments (11) to the animal gut rumen (12).

Not only Sputnik but also multiple isolated virophages also affect host nucleocytovirus replication by reducing progeny production (7, 13–17). However, the interaction between virophages and host nucleocytoviruses varies depending on the combinations. Sputnik virophages are thought to attach to the surface fibrils of host mimiviruses and be incorporated into host amoeba cells together (18). In contrast, Mavirus, a virophage of CroV, can enter host protist cells independently and wait for the corresponding giant virus infection (6, 17). Among the virophages parasitizing mimiviruses, Zamilon virophages replicate without affecting host-virus replication (16), unlike Sputnik virophages.

The expanded number of isolates and genomic sequences discovered from metagenomic data has provided novel insights into the vast diversity of this complex tripartite system. However, the molecular mechanisms underlying these microbial eukaryote-giant virus-virophage interactions remain largely unknown. In the present study, we focused on one of the tripartite systems, *Acanthamoeba castellanii*, Acanthamoeba polyphaga mimivirus (APMV), and Sputnik 3 virophage, analyzing their transcriptome dynamics. Our results strengthen the concept of virophages as viruses parasitizing other viruses and identify the disturbance of APMV transcriptional regulation caused by Sputnik infection.

## RESULTS

### Transcriptome landscape of the tripartite system

**Fig 1:**
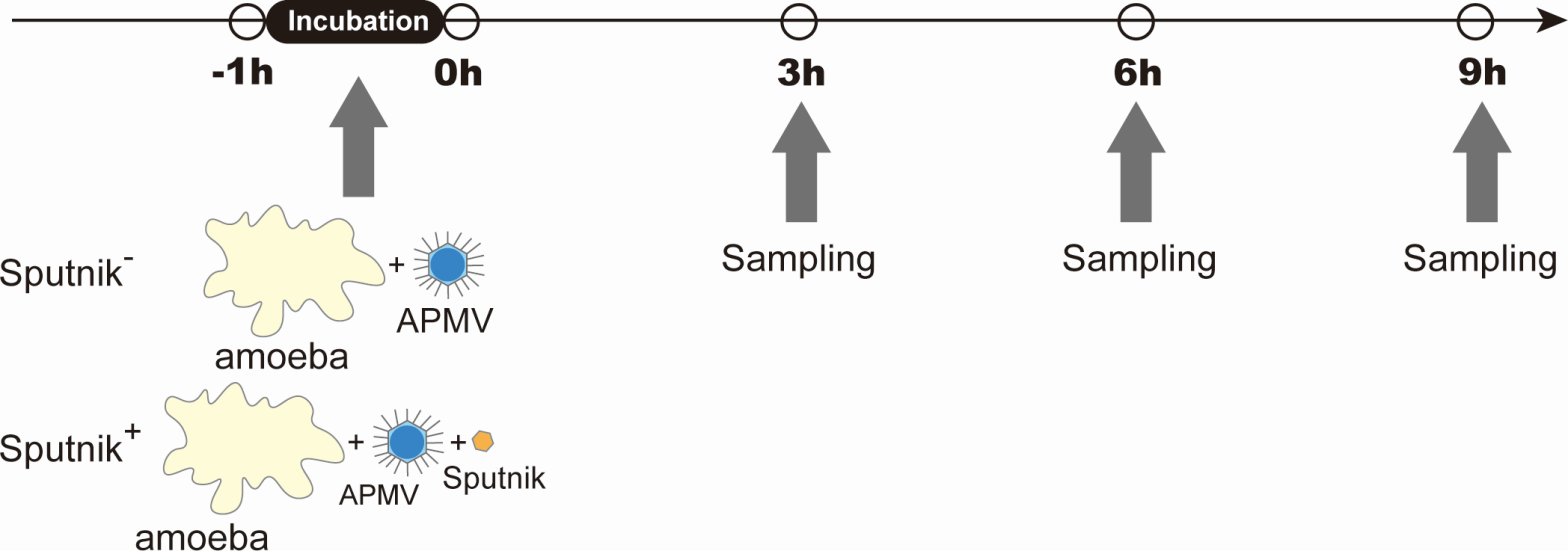
A schematic image of the experiment. Cells and viruses were incubated for 1 hour, and the supernatant was exchanged for fresh medium to remove the remaining viruses. The time when the medium was exchanged was set as 0 hpi.

Since the infection cycle of Sputnik was not fully explored, we first determined the time points for RNA sequencing based on the expression timing of marker genes: the DNA polymerase B (*polB*) gene of APMV and the major capsid protein (*mcp*) genes of both APMV and Sputnik (Fig. S1). The expression of APMV *polB* peaked at 3 hours post-infection (hpi), while the expression of APMV *mcp* peaked at 6 hpi and was maintained until 9 hpi. Sputnik *mcp* showed a similar pattern to APMV *mcp*. At 12 hpi, *polB* expression increased again, and *mcp* expression decreased, suggesting that the first infection cycle ends and secondary infection starts at this point. Based on these results, we performed transcriptome analysis at 0, 3, 6, and 9 hpi using amoeba cells infected only with APMV (Sputnik^−^ cells) and those infected with both APMV and Sputnik (Sputnik^+^ cells; Fig. 1).

**Fig 2:**
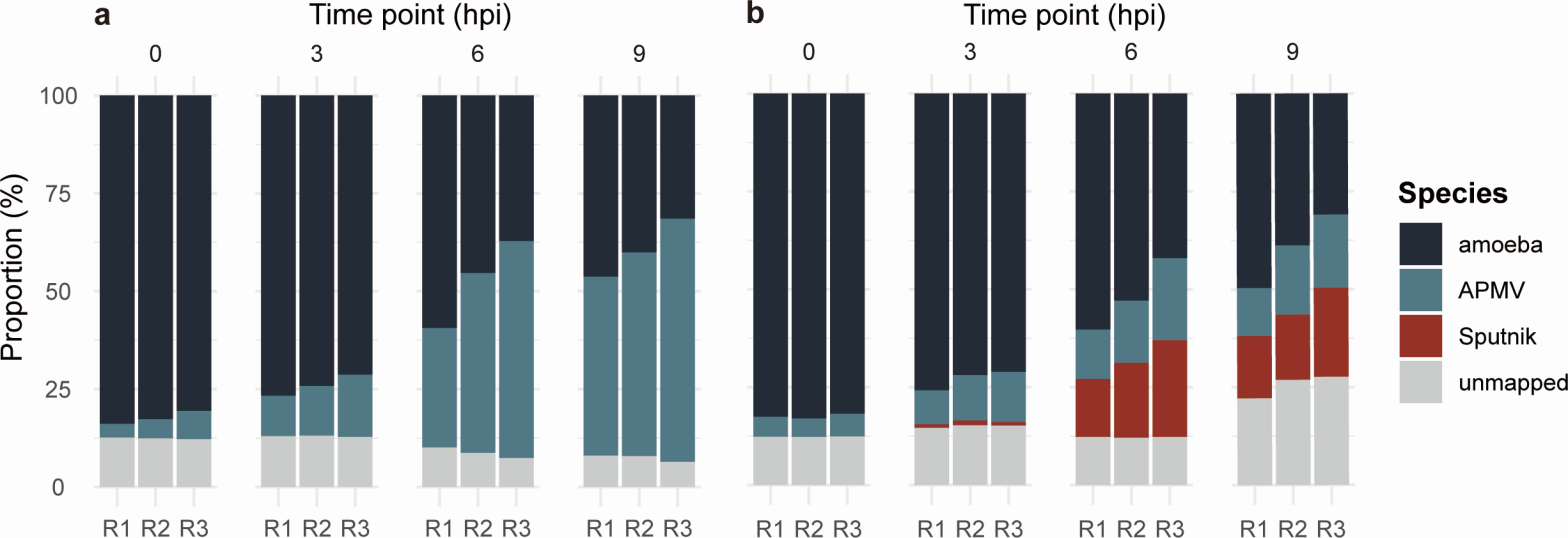
Proportions of viral and host reads at different time points. The proportion of reads from different sources relative to total reads (a) Sputnik^−^ cells, where amoeba cells were infected only with APMV. (b) Sputnik^+^ cells, where amoeba cells were infected with both APMV and Sputnik. R1, R2, and R3 represent each replicate.

In both conditions, the proportion of amoeba-derived reads decreased, while the reads from viruses increased throughout the infection (Fig. 2). In Sputnik^−^ cells, the proportion of APMV-derived reads reached approximately 50% at 9 hpi, whereas in Sputnik^+^ cells, the APMV and Sputnik reads each accounted for approximately 20% of the total reads at 9 hpi. The total proportion of viral reads in Sputnik^+^ cells was around 40%, similar to the viral-read proportion in Sputnik^−^ cells.

### Sputnik genes are divided into two groups by their expression timing

**Fig 3:**
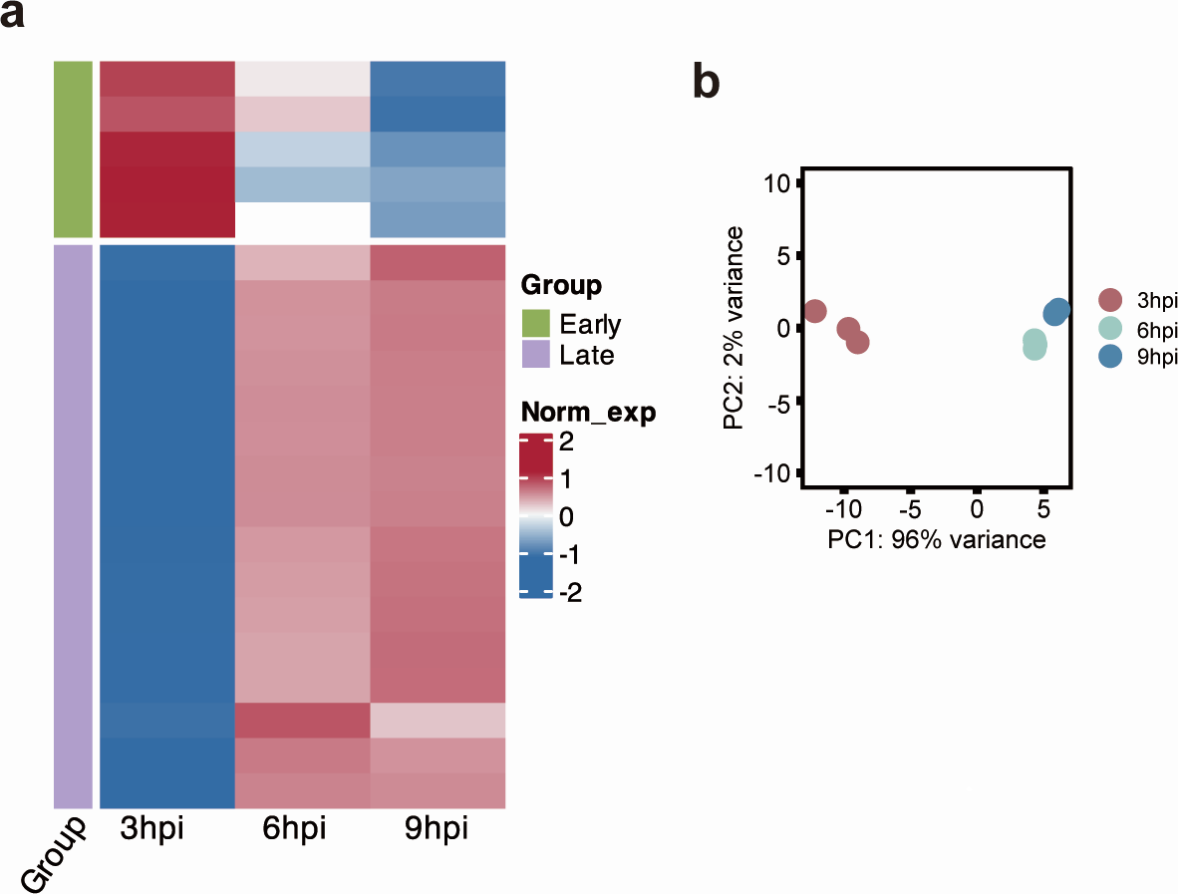
Gene expression pattern of Sputnik. (a) Heatmap and clustering based on normalized Sputnik gene expression. Gene expression was calculated as transcripts per million (TPM), followed by log2 transformation and normalized within each gene. In the left color bar, green and purple indicate early and late genes, respectively. (b) Principal component analysis (PCA) of Sputnik gene expression. Each circle represents a replicate, and each color represents a time point (*n* = 3).

As very few reads were mapped to Sputnik genes at 0 hpi (Table S1), we considered that the Sputnik gene expression at 0 hpi was likely to be an artifact. Therefore, we investigated Sputnik gene expression at 3, 6, and 9 hpi. Sputnik genes were clustered into two groups based on their expression patterns (Fig. 3). The first group, early genes (five genes), exhibited high expression at 3 hpi, gradually decreasing from 6 to 9 hpi. The second group, late genes (17 genes), had low expression at 3 hpi, but their expression increased at 6 hpi and remained at a similar level until 9 hpi (Fig. 3a). Early genes included a gene annotated with a DNA replication function, while late genes included virion-related genes such as a DNA packaging protein, a membrane protein, and capsid proteins (Table S1).

### Sputnik affects a late stage of APMV infection

**Fig 4:**
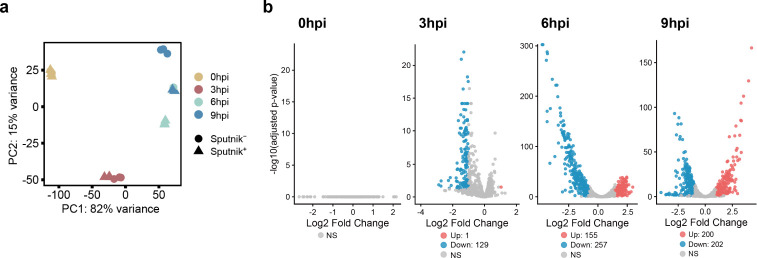
Comparison of APMV gene expression level between Sputnik^−^ and Sputnik^+^ cells. (a) PCA of APMV gene expression. Colors represent each time point, with circles and triangles representing replicates from Sputnik^−^ cells and Sputnik^+^ cells, respectively. (b) The expression changes of APMV genes are shown in volcano plots. APMV gene expression was compared between Sputnik^−^ cells (as the baseline) and Sputnik^+^ cells at each time point. Time points are indicated at the top left. Genes with an adjusted *P-*value (*P_adj_*) ≤0.05 and |log2 fold change| ≥ 1 are considered differentially expressed genes (DEGs) and are marked in red or blue. Red and blue indicate up-regulated and down-regulated genes, respectively. NS: no significant difference.

Principal component analysis (PCA) based on APMV gene expression showed that samples from Sputnik^−^ and Sputnik^+^ cells were closely located at 0 and 3 hpi (Fig. 4a). At these two time points, 0 and 130 APMV genes were differentially expressed, respectively (Fig. 4b). These results suggest similar transcription profiles of APMV at the early stages of infection between Sputnik^−^ and Sputnik^+^ cells. However, at 6 and 9 hpi, samples from Sputnik^−^ and Sputnik^+^ cells were distinctly separated (Fig. 4a). Of the 979 APMV genes analyzed, 412 (42%) and 402 (41%) genes were differentially expressed at these later time points, respectively (Fig. 4b). Moreover, samples from Sputnik^+^ cells at 9 hpi overlapped with those from Sputnik^−^ cells at 6 hpi in the PCA (Fig. 4a), suggesting that Sputnik infection inhibits the progression of APMV gene expression.

### Sputnik infection has little effect on amoeba gene expression

**Fig 5:**
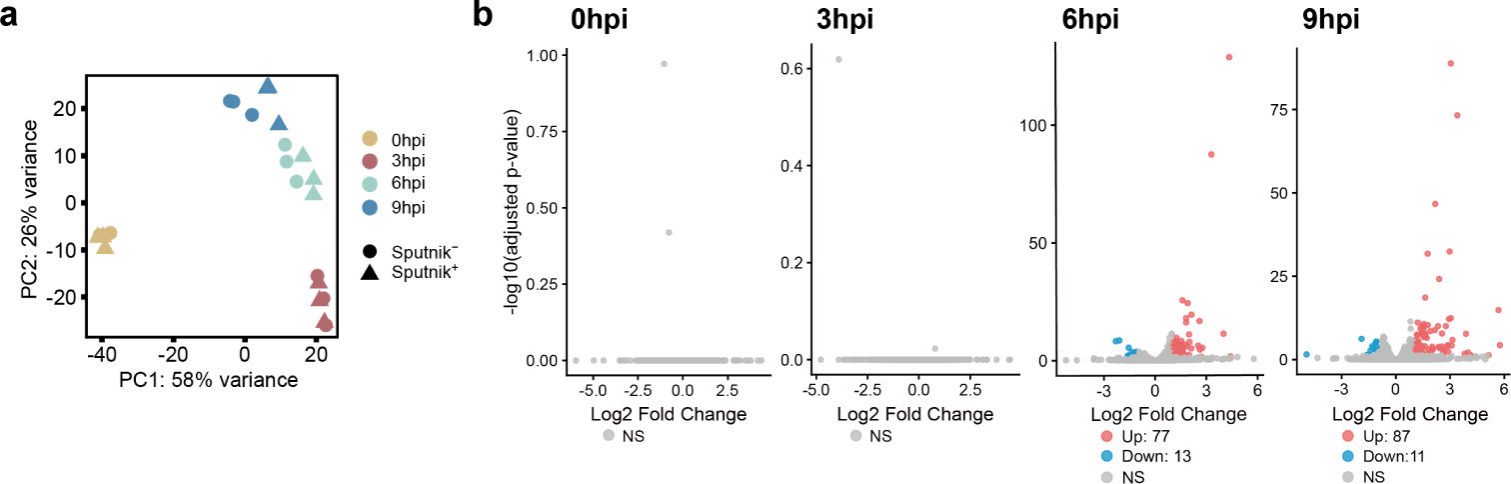
Comparison of amoeba gene expression levels between Sputnik^−^ and Sputnik^+^ cells. (a) PCA of amoeba gene expression. Circles and triangles represent each replicate from Sputnik^−^ and Sputnik^+^ cells, respectively. (b) The expression changes of amoeba genes are shown in volcano plots. Amoeba gene expression was compared between Sputnik^−^ cells (as the baseline) and Sputnik^+^ cells at each time point. Genes with *P_adj_* ≤ 0.05 and |log2 fold change| ≥ 1 are defined as differentially expressed genes (DEGs) and are marked in red or blue. Red and blue indicate up-regulated and down-regulated genes, respectively. NS: no significant difference.

In PCA based on amoeba gene expression, samples from Sputnik^−^ and Sputnik^+^ cells at each time point were closely located (Fig. 5a). No amoeba genes were differentially expressed at 0 and 3 hpi, while 90 (0.6%) and 98 (0.7%) amoeba genes were differentially expressed at 6 and 9 hpi, respectively (Fig. 5b; Table S2). Compared with the number of amoeba genes whose expression levels were altered by APMV infection (Fig. S2), these numbers were relatively small. Thus, we concluded that Sputnik infection has little effect on amoeba gene expression.

### Sputnik delays the expression of APMV genes

**Fig 6:**
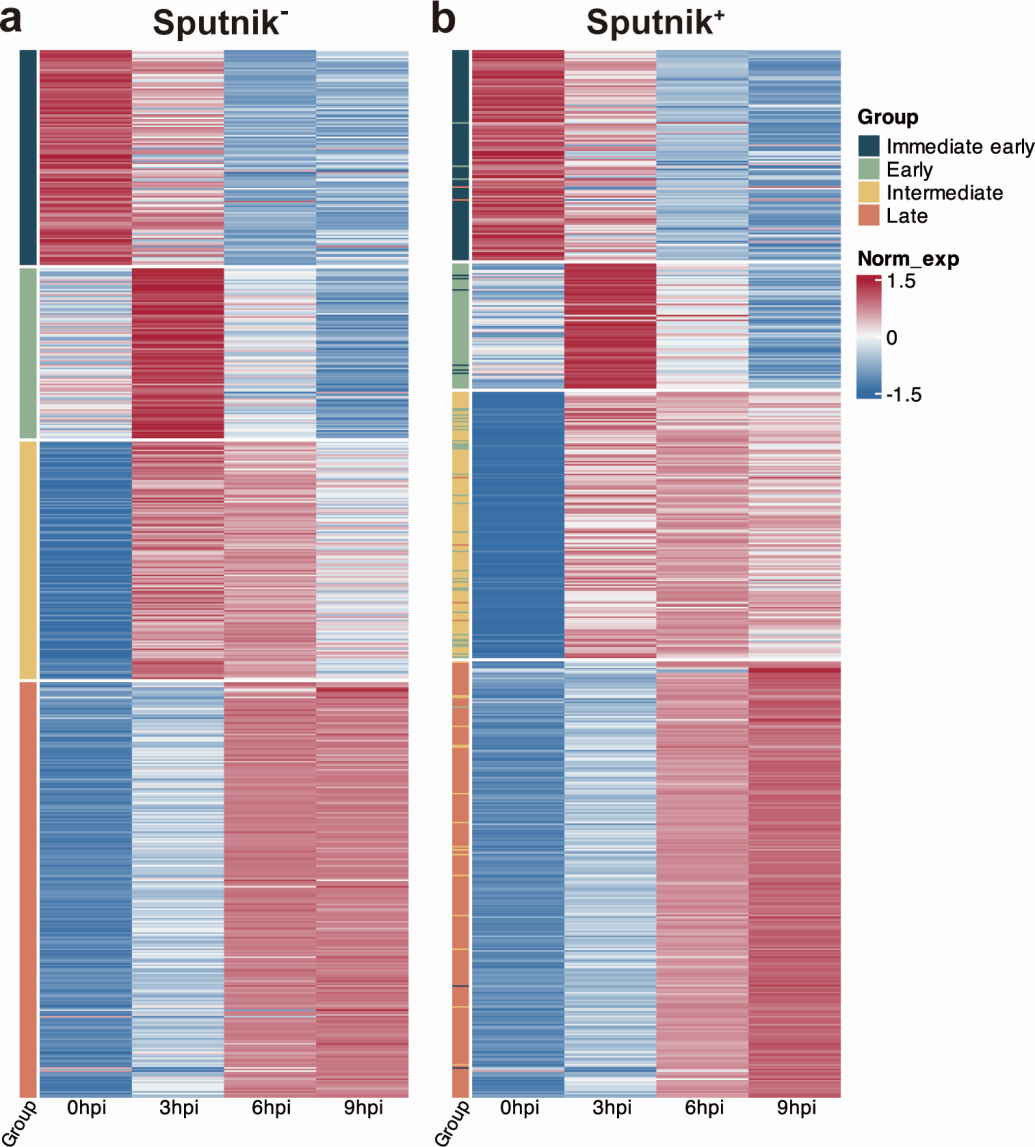
Clustering of APMV DEGs. Clustered heatmap of selected APMV DEGs from (a) Sputnik^−^ and (b) Sputnik^+^ cells. Gene expression was calculated as transcripts per million (TPM), followed by log2 transformation, and normalized within each time point. Rows and columns represent genes and time points, respectively. (a) The color bar indicates each cluster. (b) The color bar indicates the clusters identified in (a).

To elucidate the effect of Sputnik infection on APMV gene expression, we classified APMV genes based on their expression patterns in Sputnik^−^ cells. To minimize noise from genes showing minor expression changes, we focused on differentially expressed genes (DEGs) for downstream analysis (see Materials and Methods). We identified four clusters: immediate-early, early, intermediate, and late (Fig. 6a; Table S3). These clusters mostly correspond to previous classifications (19). Immediate-early genes showed high expression at 0 hpi, with a gradual decline thereafter. Early genes peaked at 3 hpi, then decreased at subsequent time points. These genes mainly include those involved in host-virus interactions and DNA replication (Fig. S3; Table S3). Intermediate genes increased in expression from 0 to 3 hpi, maintained high levels at 6 hpi, and decreased at 9 hpi. Late gene expression gradually increased from 6 to 9 hpi, and these genes are mainly involved in virion structure and morphogenesis (Fig. S3; Table S3).

We applied the same clustering method to the samples from Sputnik^+^ cells, resulting in four analogous APMV gene clusters. While most genes clustered into the analogous clusters, some genes were grouped into clusters corresponding to later stages of expression (Fig. 6b; Table S3). This result aligns with the delay in APMV gene expression observed in the PCA (Fig. 4a).

### Prolonged gene expression in immediate-early and early genes

In APMV genes clustered into analogous groups, some genes exhibited noticeably different expression patterns between Sputnik^−^ and Sputnik^+^ cells. For example, many intermediate genes showed relatively higher expression at 9 hpi in Sputnik^+^ cells compared to Sputnik^−^ cells (Fig. 6). To further examine APMV genes within each cluster, we performed subclustering based on the combined expression profiles in Sputnik^−^ and Sputnik^+^ cells.

**Fig 7:**
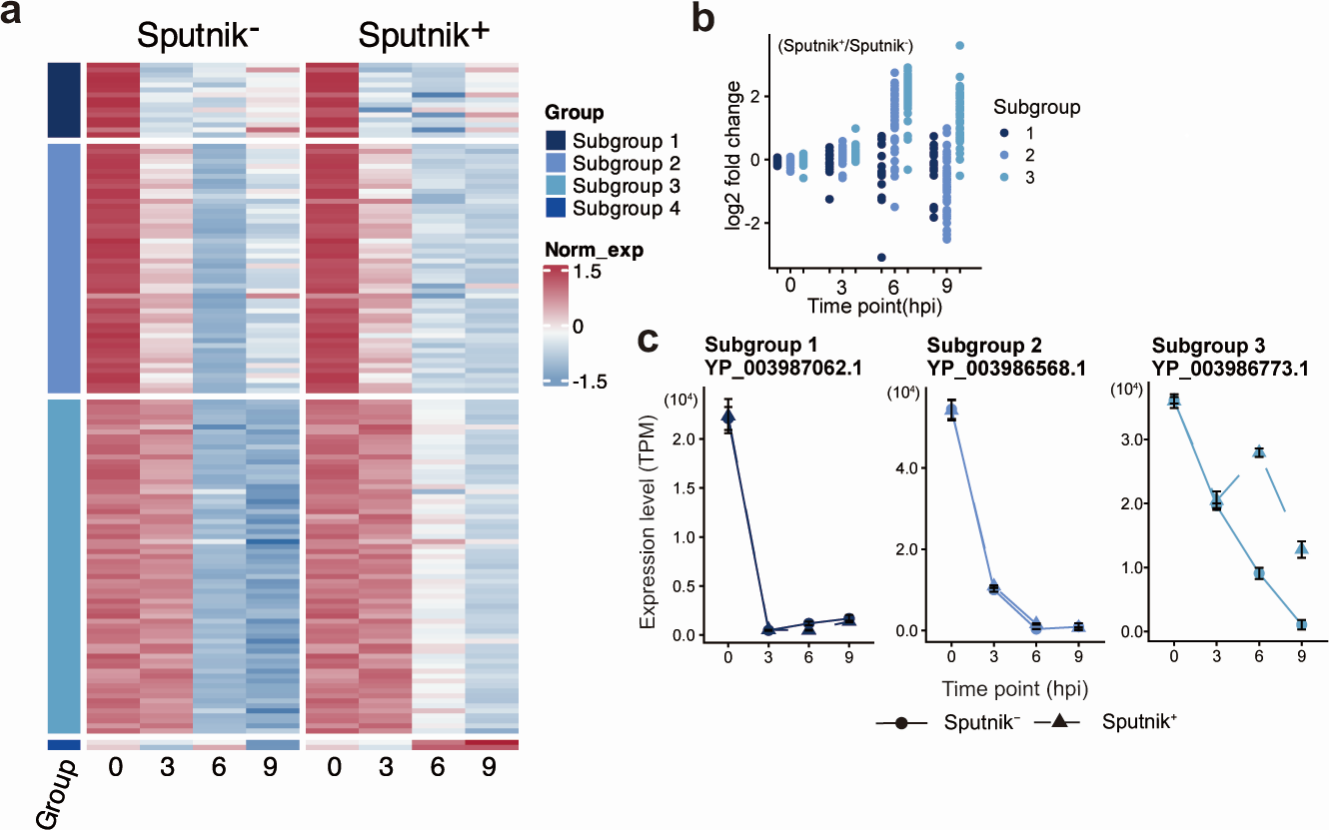
Expression profile of APMV immediate-early genes. (a) Clustered heatmap showing normalized gene expression of immediate-early genes in Sputnik^−^ and Sputnik^+^ cells. Gene expression in both Sputnik^−^ and Sputnik^+^ cells was calculated as transcripts per million (TPM) and further normalized within each gene. The color bar represents the subgroups of genes. (b) Expression changes from Sputnik^−^ to Sputnik^+^ cells at each time point. The log2-fold change of each gene expression was calculated based on TPM. Each dot represents an APMV gene. (c) Temporal expression changes of representative genes. Genes with the highest expression in subgroups 1, 2, and 3 are shown. Error bars indicate the SD (*n* = 3).

In the immediate-early genes, we identified four subgroups. However, subgroup 4 comprised two genes (Fig. 7a). Therefore, we focused on subgroups 1, 2, and 3. Genes in these subgroups exhibited comparable expression levels in Sputnik^−^ and Sputnik^+^ cells at 0 and 3 hpi. A few genes in subgroup 1, a relatively small subgroup, showed slightly decreased expression in Sputnik^+^ cells compared to Sputnik^−^ cells at 6 and 9 hpi (Fig. 7b and c). In contrast, in subgroups 2 and 3, which include most immediate-early genes, the genes showed up-regulation at 6 hpi (Fig. 7b and c). This result suggests that Sputnik infection leads to prolonged expression of these genes. At 9 hpi, this prolonged expression ceased for the genes in subgroup 2 but continued for those in subgroup 3.

**Fig 8:**
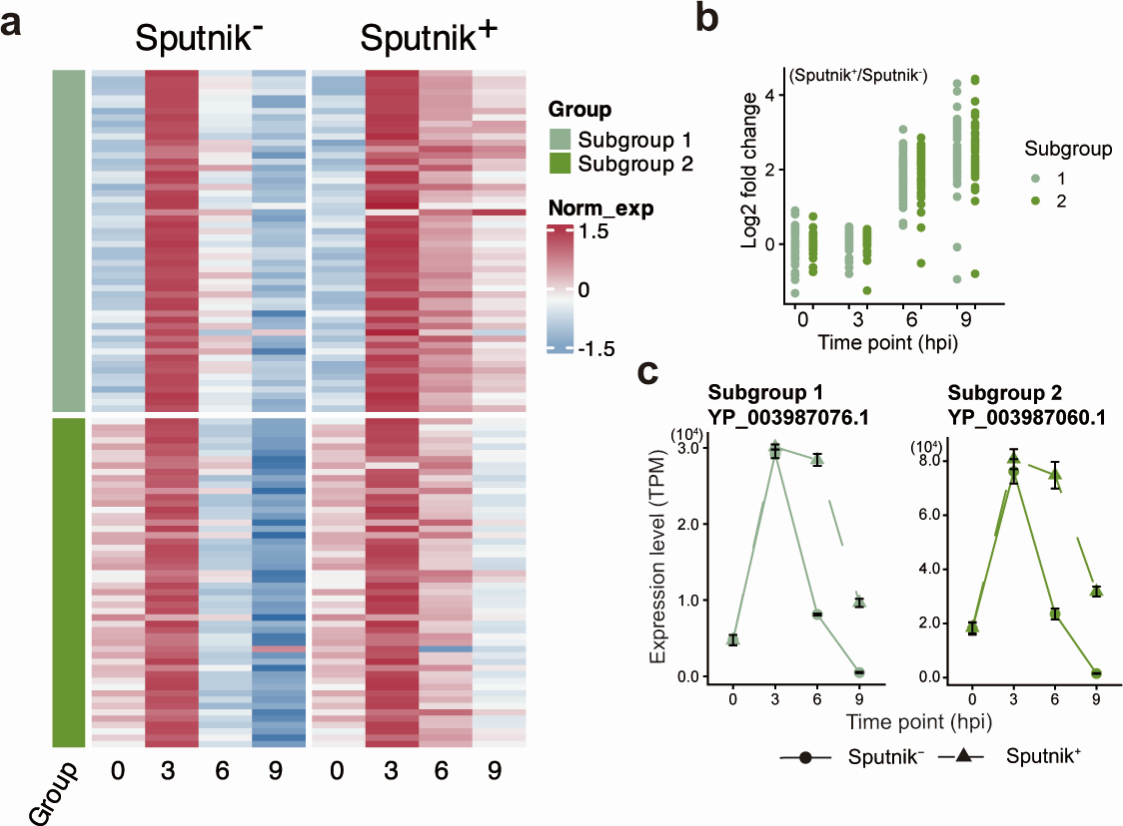
Expression profile of APMV early genes. (a) Clustered heatmap showing normalized gene expression of APMV early genes in Sputnik^−^ and Sputnik^+^ cells. Gene expression was calculated as transcripts per million (TPM), then normalized, and clustered as described in Fig. 7. (b) Expression changes from Sputnik^−^ to Sputnik^+^ cells at each time point. Each dot represents an APMV gene. (c) Temporal expression changes of representative genes. Genes with the highest expression from each subgroup are shown. Error bars indicate the SD (*n* = 3).

Early genes were divided into two subgroups (Fig. 8a). Genes in both subgroups showed similar temporal expression changes. Their expression increased from 0 to 3 hpi, with similar levels in both Sputnik^−^ and Sputnik^+^ cells. However, at 6 and 9 hpi, these genes exhibited four- to eightfold higher expression in Sputnik^+^ cells compared to Sputnik^–^ cells (Fig. 8b and c). These results suggest that Sputnik infection also prolongs the expression of APMV early genes, similar to the effect observed with a part of immediate-early genes.

### Delayed gene expression in intermediate and late genes

**Fig 9:**
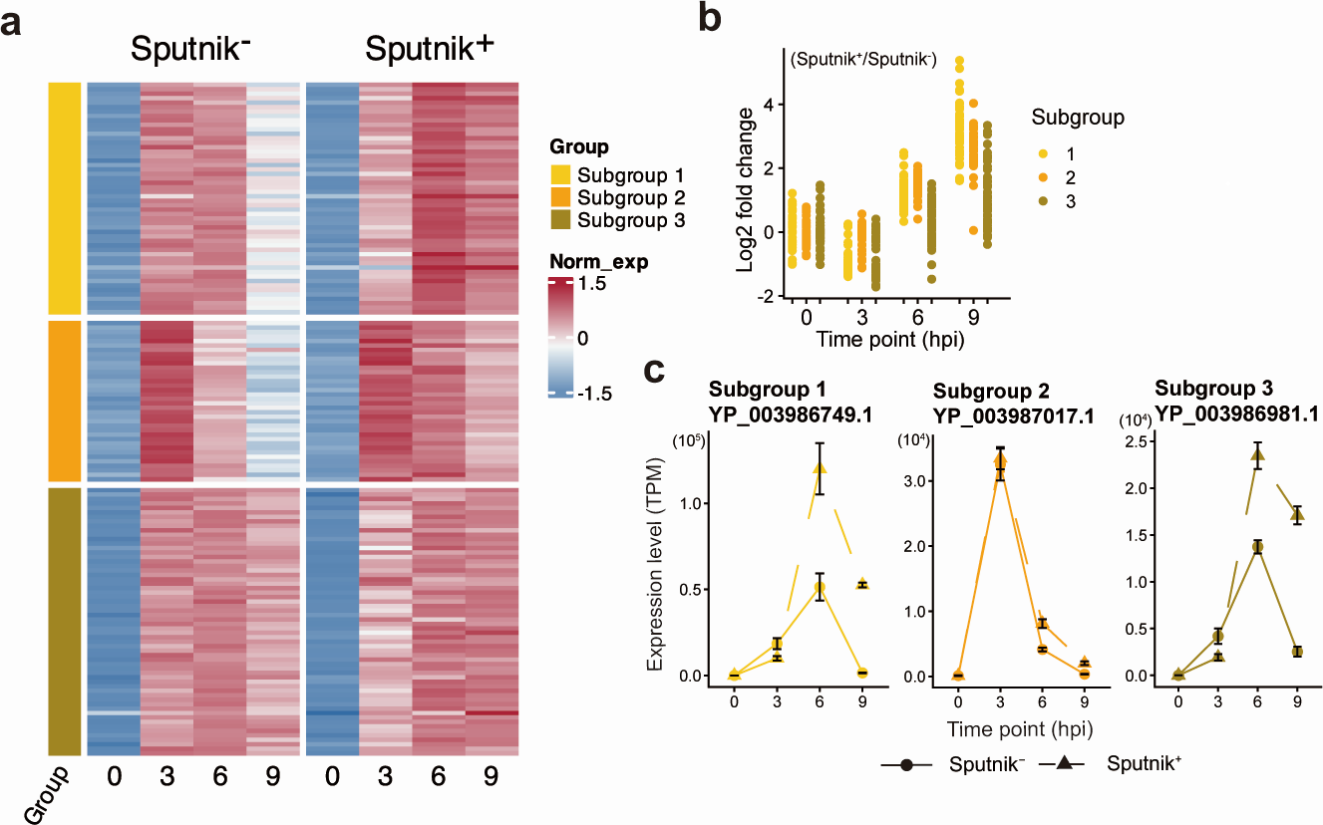
Expression profile of APMV intermediate genes. (a) Clustered heatmap showing normalized gene expression of APMV intermediate genes in Sputnik^−^ and Sputnik^+^ cells. Gene expression was calculated as transcripts per million (TPM), then further normalized, and clustered as described in Fig. 7. (b) Expression changes from Sputnik^−^ to Sputnik^+^ cells at each time point. Each dot represents an APMV gene. (c) Temporal expression changes of representative genes. Genes with the highest expression from each subgroup are shown. Error bars indicate the SD (*n* = 3).

Intermediate genes were clustered into three subgroups (Fig. 9a). Sputnik infection resulted in slightly lower expression of genes in all three subgroups at 3 hpi, but their expression reached similar or higher levels than in Sputnik^−^ cells at later stages of infection, suggesting a slight delay in the initiation of gene expression (Fig. 9b and c). Genes in subgroups 1 and 2 exhibited approximately fourfold higher expression in Sputnik^+^ cells compared to Sputnik^−^ cells at 6 hpi (Fig. 9b). Part of the genes in subgroup 3 showed lower expression in Sputnik^+^ cells than in Sputnik^−^ cells. However, the other genes in this subgroup also showed up-regulation, although the fold change was smaller than in the other subgroups. While the expression of the intermediate gene decreased at 9 hpi, the expression levels in Sputnik^+^ cells were maintained higher than that in Sputnik^−^ cells, increasing the gap in expression differences between the two conditions (Fig. 9b and c). These results suggest that intermediate genes also exhibit prolonged gene expression at late stages of infection.

**Fig 10:**
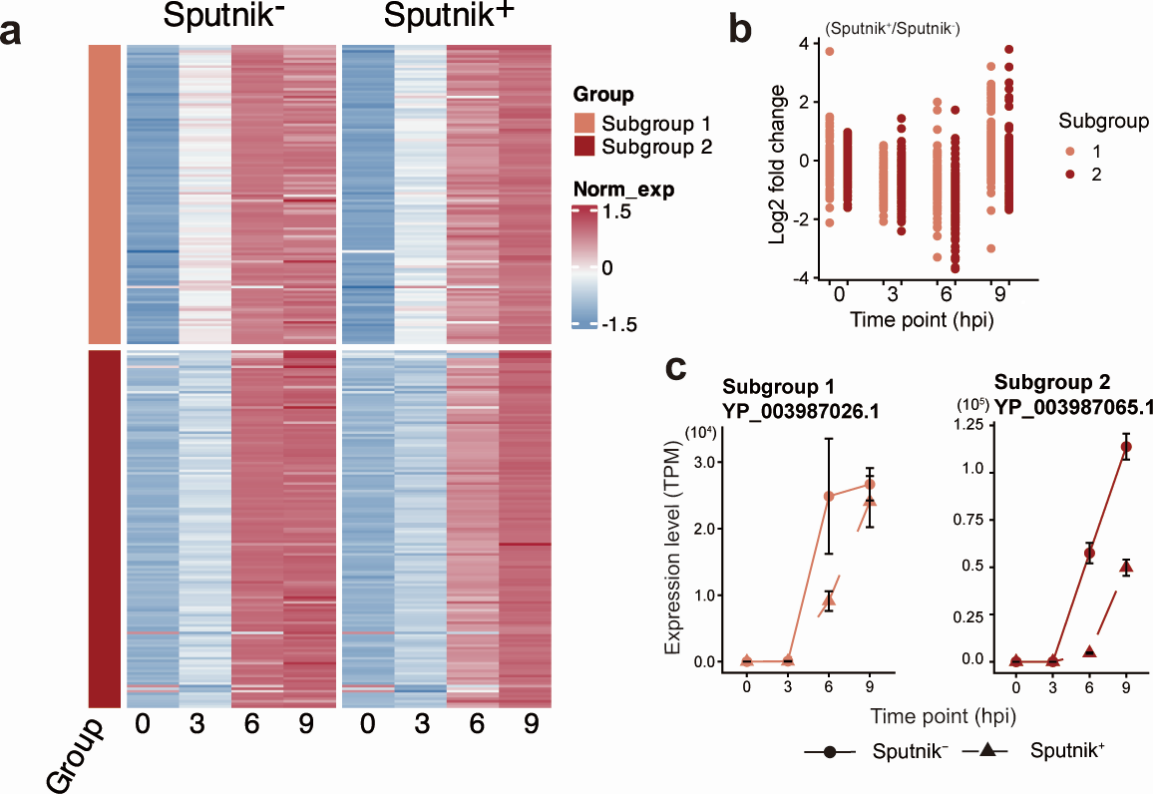
Expression profile for APMV late genes. (a) Clustered heatmap showing normalized gene expression of APMV late genes in Sputnik^−^ and Sputnik^+^ cells. Gene expression was calculated as transcripts per million (TPM), then further normalized, and clustered as described in Fig. 7. (b) Expression changes from Sputnik^−^ to Sputnik^+^ cells at each time point. Each dot represents an APMV gene. (c) Temporal expression changes of representative genes. Genes with the highest expression from each subgroup are shown. Error bars indicate the SD (*n* = 3).

Late genes were clustered into two subgroups exhibiting similar expression patterns (Fig. 10a). At 0 hpi, genes in both subgroups showed low expression in Sputnik^−^ and Sputnik^+^ cells. Most genes showed lower expression in Sputnik^+^ cells compared to Sputnik^−^ cells at 3 and 6 hpi (Fig. 10b and c). This reduced expression was partially restored at 9 hpi, with some genes showing comparable expression levels in both conditions or even higher expression in Sputnik^+^ cells than in Sputnik^−^ cells (Fig. 10b). These results suggest that Sputnik infection also delays the expression timing of APMV late genes.

### Expression of APMV transcription-related genes precedes massive onset of Sputnik transcription

**Fig 11:**
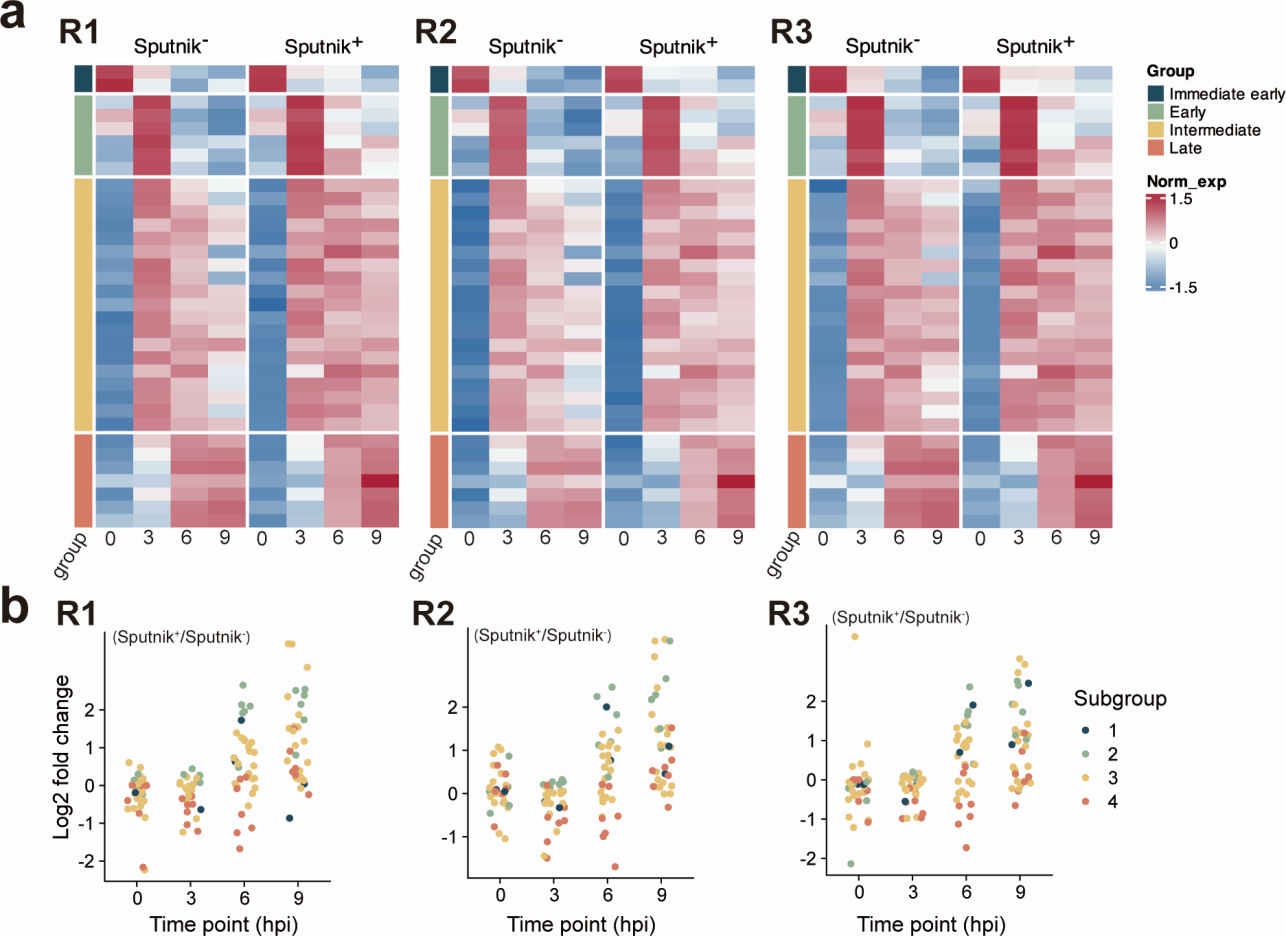
Expression of APMV transcription-related genes. (a) Clustered heatmap showing normalized gene expression of Sputnik^−^ and Sputnik^+^ cells for APMV transcription-related genes. Gene expressions were calculated as transcripts per million (TPM) and further normalized and clustered as described in Fig. 6. R1, R2, and R3 refer to each replicate. (b) Expression changes from Sputnik^−^ and Sputnik^+^ cells at each time point. Each dot represents an APMV gene. Colors represent subgroups.

As mimiviruses encode complex transcriptional machinery, we examined the expression of 34 APMV genes with transcription-related annotations (Table S4). Based on the expression patterns, these transcription-related genes were categorized into four groups by clustering (Fig. 11a). Most transcription-related genes were classified as early and intermediate genes and already expressed at 3 hpi, preceding the massive onset of Sputnik transcription (i.e., late genes; Fig. 2 and 11). These genes showed higher expression in Sputnik^+^ cells at the late stage of infection (i.e., 6 and 9 hpi), like other early and intermediate genes with prolonged expression. We also identified genes (i.e., putative cytidine deaminase and putative homeobox protein) that were classified as immediate early genes, implying these genes are involved in the expression of the early Sputnik genes.

### Promoter analysis of APMV and Sputnik genes

A previous study (19) identified an early (AAAATTGA) and late (AT-rich element) promoter of APMV genes in the upstream region of some Sputnik genes. Among five early genes, we identified two genes with both early and late promoter motifs, two with late promoter, and one without any motif. Among 16 late Sputnik genes, we identified 8 genes with late promoter, 1 with early promoter, and 7 without any motif. These results suggest that Sputnik, at least in part, uses APMV promoters for its gene expression.

**Fig 12:**
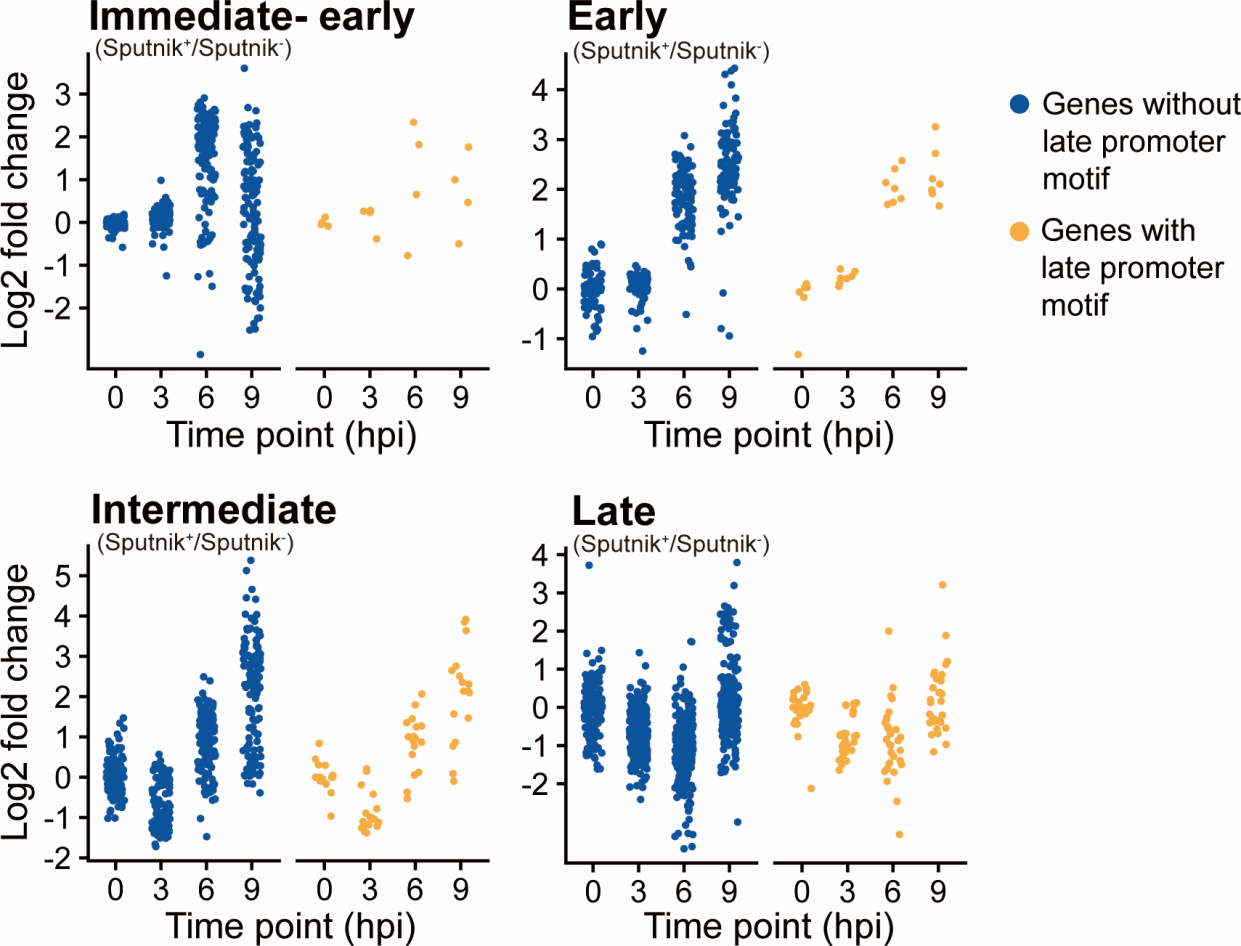
Effects of the shared promoter motif on APMV gene expression. Expression changes from Sputnik^−^ to Sputnik^+^ cells at each time point. Blue and yellow indicate genes with and without the shared promoter motif, respectively. Each dot represents an APMV gene classified as DEG.

The shared promoter between APMV and Sputnik led to the hypothesis that Sputnik uses these promoters to exploit APMV transcription machinery (19). In this case, the expression of APMV genes with the promoter may be affected. We, therefore, investigated the expression level of APMV genes with the late promoter reported in the previous study (19). Out of the 95 APMV genes with the late promoter, 60 genes were differentially expressed in this study, indicating that Sputnik disrupts the expression of the genes with the late promoter. However, the expression patterns of these 60 genes were similar to those of the differentially expressed genes without the late promoter (Fig. 12). These results suggest that the use of the late promoter by Sputnik has little effect on the differential expression of APMV genes.

We further searched for new conserved motifs in the upstream regions of the APMV genes identified as DEGs. We identified the previously reported APMV early promoter motif AAAATTGA (19), its complemented motif, and a motif similar to the late promoter but failed to find other specific promoter motifs (Fig. S4). Also, a search for motifs in the two Sputnik gene clusters revealed no statistically significant motifs (Fig. S5).

## DISCUSSION

Sputnik virophages parasitize mimiviruses and reduce their propagation (4). A recent study reported the transcriptomic landscape of cells infected with another virophage, Zamilon, which has little effect on the propagation of host mimiviruses (20). Nevertheless, the molecular mechanisms underlying the interference by Sputnik in host mimivirus propagation are largely unknown. In the present study, we investigated the transcriptome landscape of amoeba cells infected with APMV and Sputnik. We found that Sputnik infection drastically alters the APMV gene expression pattern at the late stages of infection. The PCA of the APMV gene expression profile indicated that Sputnik infection inhibits the progression of mimivirus infection from 6 to 9 hpi. The expression patterns of late and intermediate genes further support this delay. These genes exhibited reduced expression at the early stages of infection, but their expression increased at the late stages in Sputnik-infected cells as found in cells only infected with APMV. Taken together, our results demonstrate that Sputnik infection disrupts the transcriptional regulation of APMV at the late stages of infection, similar to Zamilon infection.

**Fig 13:**
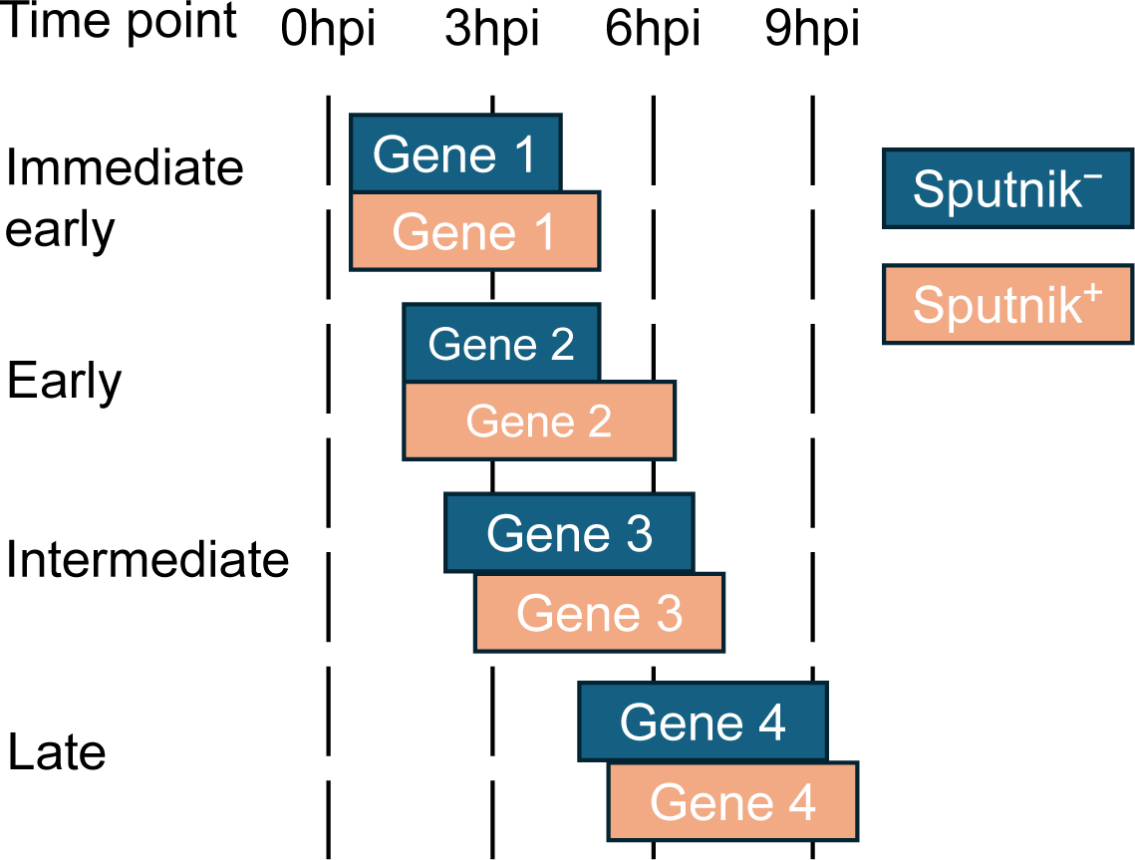
A hypothetical model of how Sputnik infection affects APMV gene expression. Each box represents a period of APMV gene expression.

Sputnik infection affects not only intermediate and late genes but also immediate-early and early genes. The expression of these genes typically starts at the early stages of infection and generally decreases at the late stages. Although these genes also showed decreased expression at the late stages in Sputnik-infected cells, the decrease was smaller compared to cells without Sputnik infection. Consequently, higher expression level was maintained at the late stages of infection in Sputnik-infected cells compared to those only infected with APMV. These results indicate that Sputnik infection prolongs the expression of APMV immediate-early and early genes. Meanwhile, this prolongation suggests that Sputnik infection may hinder the transition from the early to the late stages of APMV infection, disrupting APMV late gene expression (Fig. 13).

APMV immediate-early and early genes typically include those involved in DNA replication. Prolonged expression of these genes might benefit Sputnik, as it possesses a limited number of genes related to DNA replication. Also, transcription-related genes encoded by APMV showed similar prolonged expression, which may also benefit Sputnik gene expression. However, since most APMV genes have not been fully characterized, further studies are needed to understand the advantages of Sputnik in disturbing APMV gene expression.

Virophage was named after bacteriophage because its lifecycle depends on a host giant virus as bacteriophages depend on a host bacterium (4). This study revealed that Sputnik gene expression begins following APMV gene expression, including APMV transcription-related genes. We also found that Sputnik infection has little effect on both the proportion of amoeba-derived reads and the amoeba gene expression pattern. Combined with the recent study using Zamilon virophage (20), our results indicate that mimiviruses are primary hosts of Sputnik, supporting the original concept of virophage.

A previous study found promoter motifs that are shared between Sputnik and APMV genes and indicated that using the same promoter is a potential mechanism for virophages to exploit the transcriptional machinery of mimiviruses (19). Our results suggest that Sputnik partially uses the APMV promoter motifs for its gene expression. However, the Sputnik infection disrupts APMV gene expression independent of the presence of the promoter. We also searched for additional promoter motifs for APMV but failed to detect these motifs. These results suggest that the shared promoter between APMV and Sputnik is not the main mechanism underlying the transcriptional changes in the APMV genes. Instead, we found that expression changes caused by Sputnik varied even among genes expressed at a similar timing. Although we failed to identify candidate genes, Sputnik might hijack specific transcriptional machinery to get precise manipulation of APMV genes. Combined with recent findings from other model systems of host giant virus-virophage interactions (17, 20), future studies may provide insights into the molecular mechanisms underlying this complex tripartite system.

## MATERIALS AND METHODS

### Cells and viruses

*A. castellanii* (Douglas) Page, strain Neff (ATCC 30010), was maintained in peptone-yeast extract-glucose (PYG) medium at 28°C. APMV and Sputnik 3 (4) were served as prototypes of mimivirus and virophage, respectively.

### Titration of viruses

The titer of APMV was measured using the 50% tissue culture infectious dose (TCID_50_) method on a 96-well plate. The titer of Sputnik was determined using a modified TCID_50_ method. Briefly, amoeba cells were infected with APMV at a multiplicity of infection (MOI) of 1 on a 96-well plate, followed by inoculation with a serially diluted Sputnik solution. Wells containing infectious Sputnik were initially inferred by observing a reduced cytopathic effect under light microscopy compared to wells containing only APMV. The presence of Sputnik was confirmed by quantitative PCR (qPCR) as follows: the entire volume of supernatant and remaining cells was collected from wells at the highest dilution level containing infectious Sputnik, as well as from one order higher and lower dilution levels. Viral DNA was extracted from these wells using the following method: the collected supernatant was centrifuged at 15,000 rpm (Thermo Scientific, Sorvall ST 8FR centrifuge) for 1 hour at 4°C. The pellets were resuspended in 45 µL of 50 mM sodium hydroxide solution and incubated at 95°C for 5 minutes. Then, 5 µL of 1 M Tris(hydroxymethyl)aminomethane hydrochloride (pH 8.0) and 450 µL Tris-EDTA buffer (10 mM Tris, 1 mM EDTA, and pH8.0) were added to the solution, which was then subjected to qPCR. The reaction mixture was prepared using the KAPA PROBE Fast qPCR kit (ROXLowqPCR, KAPA BIOSYTEMS), with 0.3 µM forward and reverse primers and 0.4 µM probe. The PCR conditions were as follows: 95°C for 3 min; 40 cycles of 95°C for 3 s, 60°C for 20 s, and 72°C for 1 s. The sequences of the primers and probe (21) are shown in Table S5.

### Sample preparation

A total of 1 × 10^6^ amoeba cells were inoculated with APMV or APMV and Sputnik at an MOI of 10 in PYG medium. The inoculated cells were incubated for 1 hour at room temperature with gentle shaking. After incubation, the culture medium was replaced with 1 mL of phosphate-buffered saline (PBS) to remove uninfected viruses. The PBS was then replaced with 1.5 mL of fresh PYG medium, and the cells were incubated at 30°C for the designated periods. The time point at which the medium was exchanged for fresh PYG medium was designated as 0 hpi.

### RNA extraction

Infected cells were collected at 0, 1, 3, 6, 9, and 12 hpi using a cell scraper (Thermo Fisher Scientific) and then centrifuged at 2,000 rpm for 5 min at 4°C. The cell pellet was stored in 1 mL of TRIzol (Invitrogen) at −80°C. Three independent experiments were conducted as biological replicates. RNA extraction followed the manufacturer’s protocol. Briefly, 20% of the sample volume of chloroform was added to the cells in TRIzol. The RNA-containing aqueous layer was transferred to new tubes to which 500 µL of isopropanol was added to precipitate RNA. The RNA pellet was washed with 70% ethanol, dissolved in nuclease-free water, and treated with DNase I (New England Biolabs). RNA was then re-extracted using phenol/chloroform and re-precipitated with 99.5% ethanol and 0.3 M sodium acetate. The precipitate was washed with 70% ethanol and dissolved in nuclease-free water. RNA concentration was measured using a Qubit 4 Fluorometer (Invitrogen) with the Qubit RNA BR Assay Kit (Invitrogen). RNA purity was assessed by measuring the A260/A280 and A260/A230 ratios using a DeNovix DS-11 spectrophotometer (SCRUM Inc.).

### RNA sequencing

Quality control and sequencing of RNA samples were performed by Rhelixa Inc. (Japan). In brief, strand-specific libraries were prepared using poly-A selection and sequenced to a depth of 5 G bases per sample using Illumina NovaSeq 6000.

### Measuring gene expression by qPCR

Extracted RNA was adjusted to a uniform concentration across all samples using nuclease-free water. For cDNA synthesis, 0.5 µg of total RNA was used with D(T)23VN primer (New England Biolabs), 10 mM dNTPs (New England Biolabs), AMV reverse transcriptase (New England Biolabs), and an RNase inhibitor (New England Biolabs). The reaction was conducted at 42°C for 1 hour, followed by enzyme deactivation at 80°C for 5 min.

The synthesized cDNA was then subjected to qPCR using the KAPA SYBR Fast qPCR kit (ROXLowqPCR, KAPA BIOSYTEMS) with 0.2 µM of forward and reverse primers. The sequences of primers (21, 22) are listed in Table S5. The PCR conditions were as follows: 95°C for 20 s; 40 cycles of 95°C for 15 s, 60°C for 20 s, and 72°C for 10 s. Relative gene expression was calculated using the 2^ΔCt^ method, with the highest expression level set as 100.

### Data analysis

The quality of reads was assessed using FastQC (v0.12.0) (23). With an overall quality score exceeding 20 and no known adaptors detected, no trimming was performed. Reads were mapped to a sequence data set consisting of the Sputnik 3 genome (NC_011132.1) and the APMV genome (NC_014649.1) using HISAT2 (v 2.2.1) (24), with a maximum intron size of 5,000 bp. Unmapped reads were subsequently mapped to the *A. castellanii* genome (GCF_000313135.1_Acastellanii.strNEFF_v1) with a maximum intron size of 500,000 bp. Output data were processed with Samtools (v1.19) (25). The number of reads mapped to each gene was counted using HTSeq (v2.0.5) (26) in union mode with the reverse strand-specific assay option. Gene expression levels were individually normalized to transcripts per million (TPM) by using summed reads mapped to Sputnik, APMV, and amoeba genome.

PCA and DEG detection were conducted using DESeq2 (1.42.0) (27) with read count data for each gene. Genes were classified as DEGs with a false discovery rate <0.05 and absolute log2-fold change ≥1 at any time point (28).

DEGs were clustered using the k-means method. Log2-transformed TPM for each gene was normalized by adjusting the mean to 0 and the variance to 1 before clustering. The optimal number of clusters for Sputnik and APMV was determined using the total-within-sum-of-squares method in factoextra (v1.0.7).

Annotation information for APMV genes was retrieved from the National Center for Biotechnology Information GenBank database. Functional categories for each APMV gene were assigned manually, referring to previous studies (19, 29–31).

### Promoter search

One hundred base pair sequences upstream of the open reading frames of each DEG in Fig. 5 matching the same expression timing were extracted. Sequence motifs were analyzed using MEME Suite 5.5.4 (32). Previously identified motifs were searched by FIMO using settings in a previous study (19). Also, sequence motifs were predicted *de novo* by MEME. MEME was run in classic mode with motif width ranges set from 8 to 25 bp and the “any number of repetitions” option.

### Visualization

All data processing was carried out using the dplyr (v1.0.10) (33) and tidyr (v1.3.0) (34) packages in R (v4.2.1) (35). Heatmaps were generated with the pheatmap (v1.0.12) package (36). All plots were created using the ggplot2 (v3.4.4) package (37).

## Data Availability

The RNA sequencing data are available at NCBI/ENA/DDBJ under accession number PRJDB18584.

## SUPPLEMENTAL MATERIAL

Supplemental material jvi.00192-25-s0001.docx Figures S1 to S5; Table S5.

Additional supplemental tables jvi.00192-25-s0002.xlsx Tables S1 to S4.

ASM does not own the copyrights to Supplemental Material that may be linked to, or accessed through, an article. The authors have granted ASM a non-exclusive, world-wide license to publish the Supplemental Material files. Please contact the corresponding author directly for reuse.
