# Supplementary material for: Sputnik virophage disrupts the transcriptional regulation of its host giant virus
Source: J Virol. 2025 Mar 12;99(4):e00192-25. doi: 10.1128/jvi.00192-25 (PMC11998528; doi:10.1128/jvi.00192-25)
Supplement: Supplemental material — Figures S1 to S5; Table S5. [file jvi.00192-25-s0001.docx]

**Supplementary information**

**Sputnik virophage disrupts transcriptional regulation of its host giant virus**

Jingjie Chen^a^, Hiroyuki Ogata^a^, Hiroyuki Hikida^a#^

^a^Bioinformatics Center, Institute for Chemical Research, Kyoto University, Gokasho, Uji, 611-0011, Japan

^#^Corresponding author: hhikida@scl.kyoto-u.ac.jp

**Fig. S1–S5**

*** Table S1. Counts of reads mapped to the Sputnik genome**

*** Table S2. Differentially expressed host genes**

*** Table S3. APMV genes with assigned clusters and annotations**

*** Table S4. Expression (TPM) of APMV transcriptional genes with assigned clusters and annotations**

**Table S5. List of primers**

**References**

(*Included in a separate Excel file.)

**
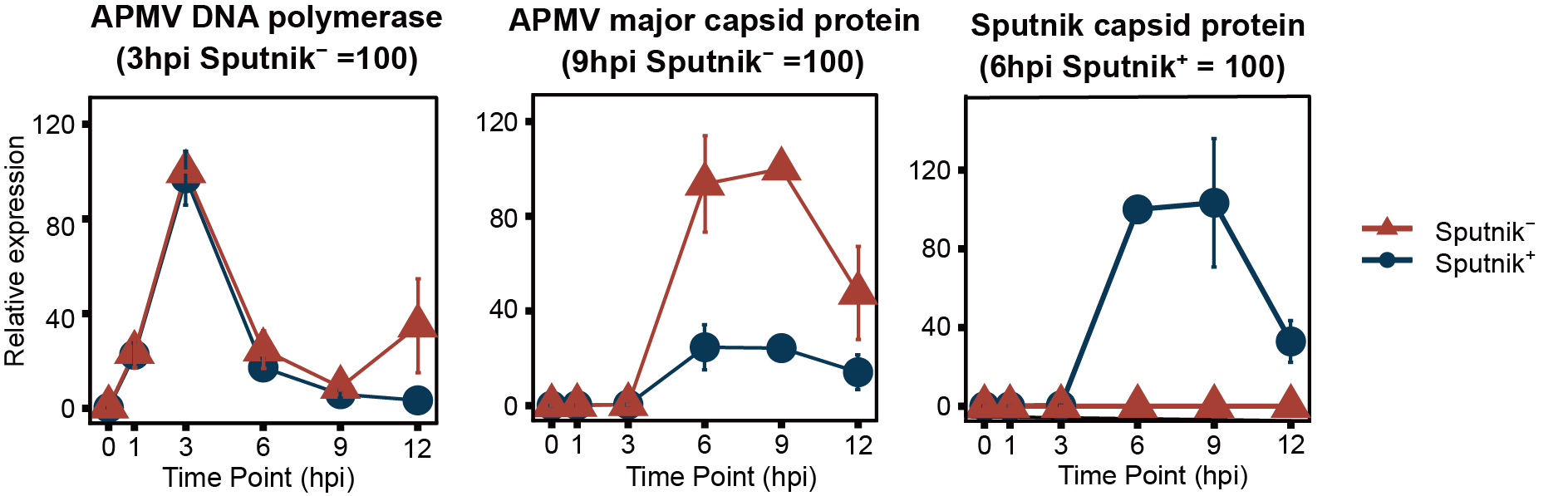
**

**Fig S1. Expression profiles of representative APMV and Sputnik genes**.

Relative expression of representative genes was measured by quantitative polymerase chain reaction. Error bars represent the standard error (n = 3). Expression levels are normalized as follows: 100 at 3 hpi for APMV DNA polymerase in Sputnik^−^ cells, 100 at 9 hpi for APMV major capsid protein in Sputnik^−^ cells, and 100 at 6 hpi for Sputnik major capsid protein in Sputnik^+^ cells.


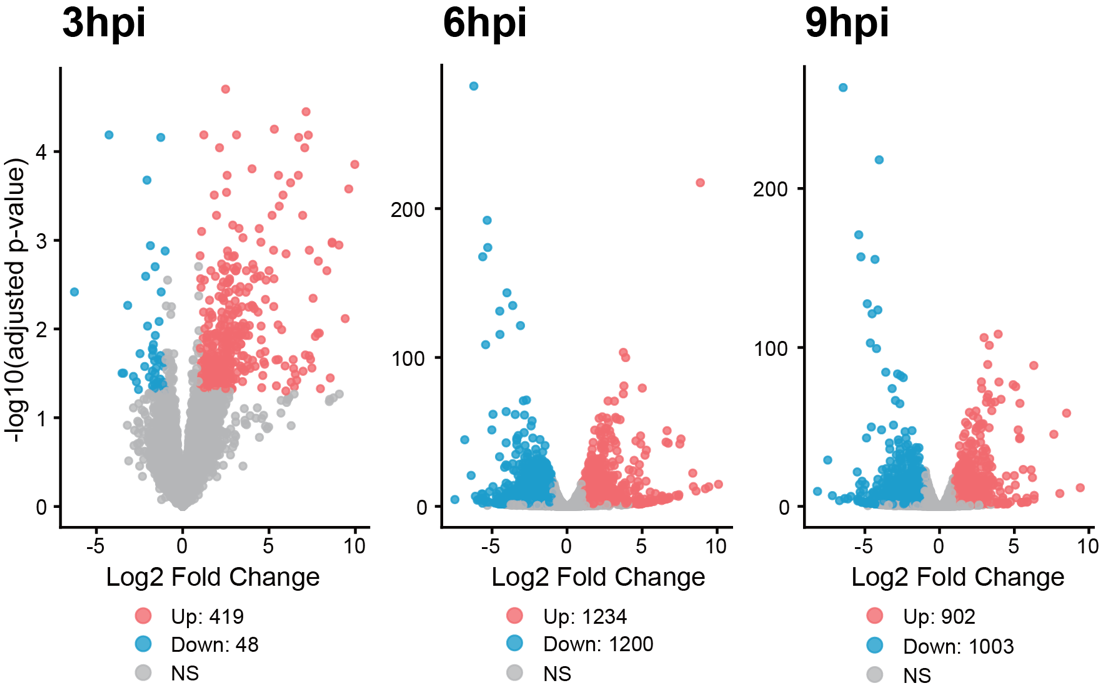


**Fig S2. Comparison of amoeba gene expression in Sputnik^−^ cells over time.**

Gene expression in Sputnik^−^ cells at 3, 6, and 9 hpi was compared to expression at 0 hpi. Genes with *p_adj_* ≤ 0.05 and |log2 fold change| ≥ 1 are considered differentially expressed genes and are marked in red or blue. Red and blue represent up-regulated and down-regulated genes, respectively. NS: no significant difference.


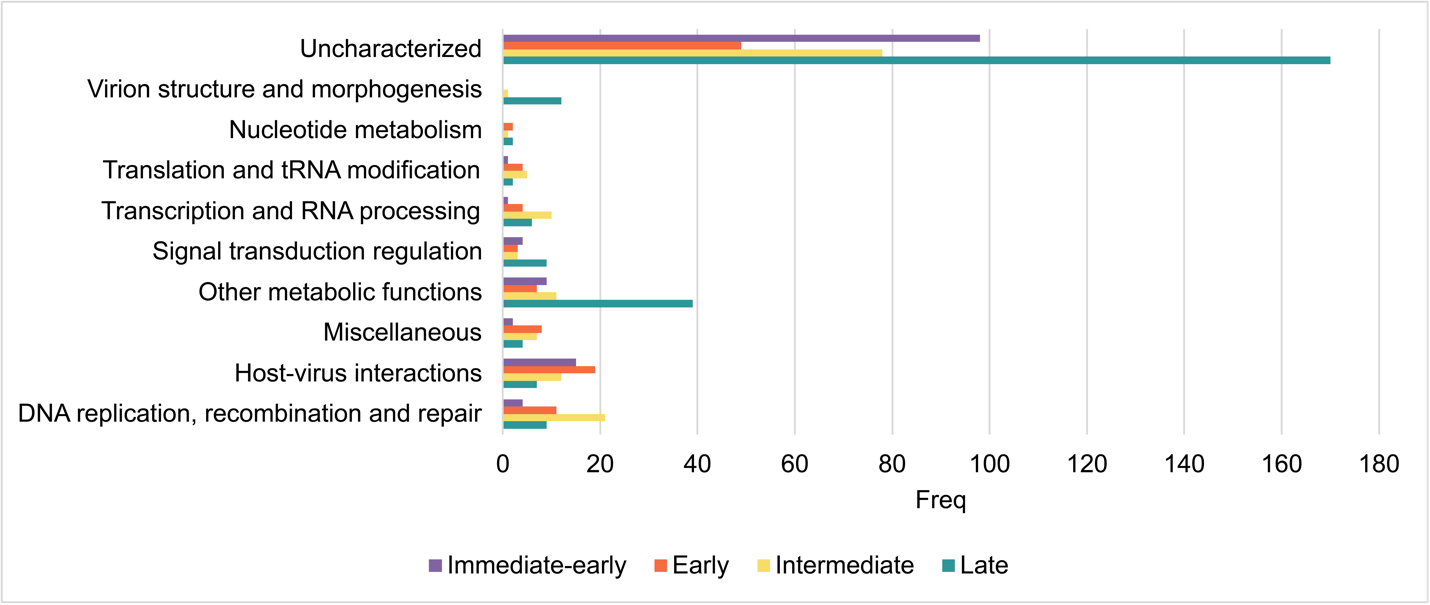


**Fig S3. Functional annotations of APMV genes across different clusters.**

The number of genes assigned to each functional category is displayed. Each color indicates the timing of gene expression. Functional categories for each APMV gene were manually assigned based on previous studies^1–5^.

**
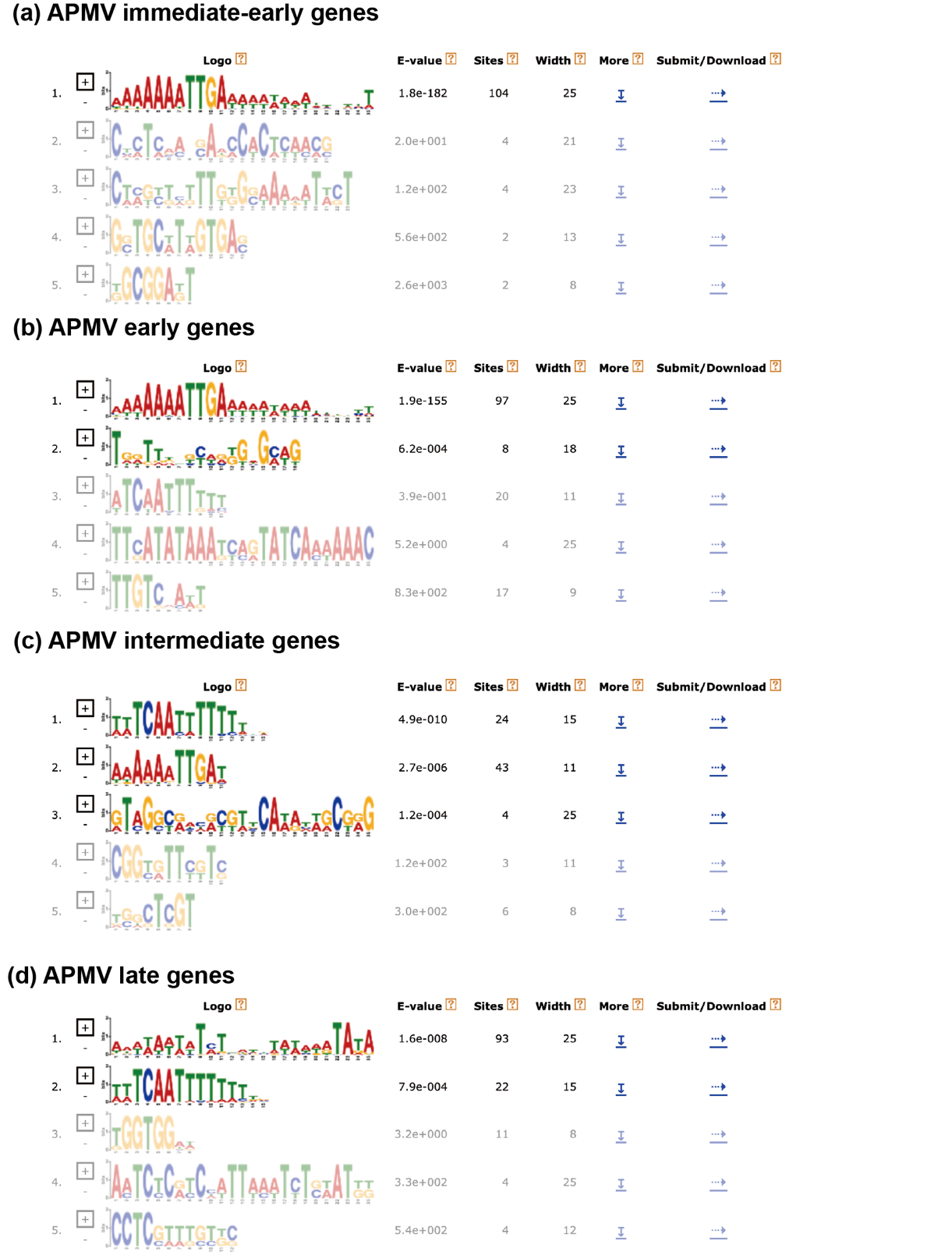
**

**Fig S4. Promoter analysis of APMV using MEME.**

Predicted motifs in (a) immediate-early, (b) early, (c) intermediate, and (d) late genes with differential expression (false discovery rate < 0.05 and |log2-fold change| ≥ 1). Motifs that met the significance threshold (E-value < 0.05) are highlighted in bright colors.


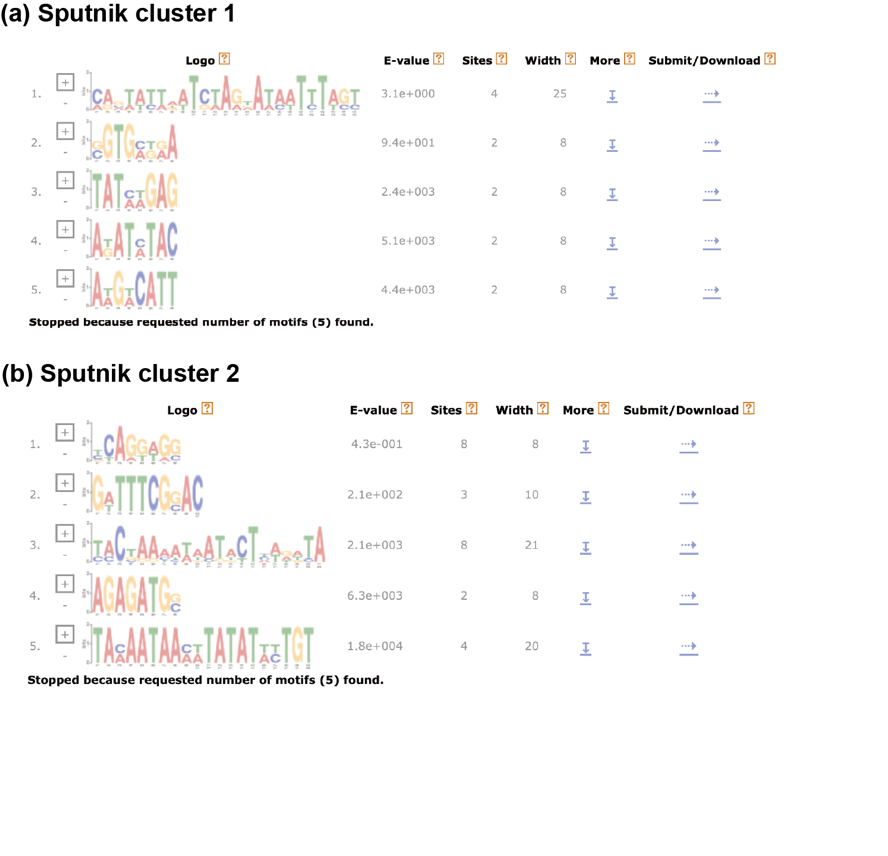


**Fig S5. Promoter analysis of Sputnik using MEME.**

Predicted motifs for (a) early and (b) late Sputnik genes. No motifs met the significance threshold (E-value < 0.05).

**Table S5. List of primers.**

| **Target** | **Sense** | **Sequence (5’ → 3’)** |
| --- | --- | --- |
| Sputnik capsid protein | Forward | GAGATGCTGATGGAGCCAAT |
|  | \|  \| \| --- \|   Reverse | CATCCCACAAGAAAGGAGGA |
| APMV DNA polymerase | Forward | TGCGGGAGTTGGAGAAATGATTGTC |
|  | Reverse | TTGGCAGCCCTTTGACACTTC |
| APMV major capsid protein | Forward | GAACCTGGAGGTTATGAATGTGAAGG |
|  | Reverse | ACCATCGAAAGCTTCAGCAGTGG |
| Sputnik probe |  | TACTTCAGCAGCTGGTCTTTCTGA |

**References**

1. Boyer, M. *et al.* Mimivirus shows dramatic genome reduction after intraamoebal culture. *Proc. Natl. Acad. Sci.* 108, 10296–10301 (2011).
2. Legendre, M. *et al.* mRNA deep sequencing reveals 75 new genes and a complex transcriptional landscape in Mimivirus. *Genome Res.* 20, 664–674 (2010).
3. Tatusov, R. L. *et al.* The COG database: an updated version includes eukaryotes. *BMC Bioinformatics* **4**, 41 (2003).
4. Koonin, E. V. & Yutin, N. Origin and Evolution of Eukaryotic Large Nucleo-Cytoplasmic DNA Viruses. *Intervirology* **53**, 284–292 (2010).
5. Yutin, N., Wolf, Y. I., Raoult, D. & Koonin, E. V. Eukaryotic large nucleo-cytoplasmic DNA viruses: Clusters of orthologous genes and reconstruction of viral genome evolution. *Virol. J.* **6**, 223 (2009).
